# Ego identity development in physicians: a cross-cultural comparison using a mixed method approach

**DOI:** 10.1186/1756-0500-5-249

**Published:** 2012-05-23

**Authors:** Tanya N Beran, Efrem Violato, Sonia Faremo, Claudio Violato, David Watt, Deidre Lake

**Affiliations:** 1 Medical Education and Research Unit, University of Calgary, Faculty of Medicine, 3330 Hospital Dr. N.W., Calgary, AB, Canada

## Abstract

**Background:**

The purpose of this study was to examine the career decision-making process of International Medical Graduates (IMGs). There are two main types of IMGs who apply for licensure in Canada. Canadian International Medical Graduates (CIMGs) were Canadian citizens before leaving to study medicine in a foreign country, in comparison to those non-CIMGs who had studied medicine in a foreign country before immigrating to Canada. Given that their motivations for becoming a doctor in Canada may differ, it is important to examine how they decided to become a doctor for each group separately.

**Methods:**

A total of 46 IMGs participated in a semi-structured interview - 20 were CIMGs and 26 were non-CIMGs.

**Results:**

An iterative process of content analysis was conducted to categorize responses from five open-ended questions according to the Ego Identity Statuses theory of career decision-making. Event contingency analysis identified a significant difference between CIMGs and non-CIMGs, Fisher’s exact test (1) = 18.79, *p* < .0001. A total of 55% of CIMGs were categorized as identity achieved and 45% as foreclosed; 100% of non-CIMGs were classified as identity foreclosed.

**Conclusion:**

About half of the Canadian citizens who had studied medicine in a foreign country had explored different careers before making a commitment to medicine, and half had not. No IMGs, however, who studied medicine in another country before immigrating to Canada, had explored various career opportunities before selecting medicine.

## Background

As a result of increased professional migration, a large number of doctors are seeking licensure in a country other than where they were educated [1]. Known as International Medical Graduates (IMGs), they comprise approximately one quarter of the physician workforce in countries such as Canada, the U.S., the U.K., and Australia [2]. Despite their demonstrated competence and value to society, they are faced with many challenging assessment procedures and limited practice opportunities, which reportedly may result in alienation and isolation [3-5]. To facilitate their integration into the profession while ensuring that they meet standards of competence, it is critical that we understand how they develop their professional identity. The purpose of the present study was to identify how each group of IMGs decided to become doctors while they were applying for certification.

### International medical graduates

There are two main types of IMGs who apply for licensure: Those who were local citizens before leaving to study medicine in a foreign country, and those who had studied medicine in a foreign country before immigrating. In Canada, the former group is known as Canadian International Medical Graduates (CIMGs), and the latter throughout this paper are referred to as non-CIMGs. Motivations for seeking licensure in Canada may differ between these groups, but this has not yet been empirically examined. It has been suggested that Canadian citizens may acquire their medical degrees outside of Canada for various reasons such as the inability to gain acceptance in a Canadian medical school, interest in experiencing life in a foreign country, or the desire to visit a country of their family’s origin. Having obtained their degree, they then wish to return to Canada to establish a long-term career in medicine. Non-CIMGs, in contrast, have obtained their medical degree in their country of citizenship, and move to Canada to practice medicine for reasons such as employment opportunities, safety, political stability, and quality of life [6,7]. The procedures for licensure in Canada are similar for both groups.

To practice medicine in Canada, applicants must complete a series of assessments and residency experience. These requirements are established by several medical organizations including the Royal College of Physicians and Surgeons of Canada, the Medical Council of Canada, College of Family Physicians, and the provincial licensing authorities (e.g. College of Physicians and Surgeons of Ontario), as well as doctor organizations such as the Canadian Medical Association. Generally, the medical school of graduation must be one that is included in the World Health Organization’s directory of medical institutions, and the medical degree must be verified by the Educational Commission for Foreign Medical Graduates International Credentials Services. Once qualifications have been verified, applicants must first pass the Medical Council of Canada Qualifying Examination Part 1. Upon demonstrating proficiency in English, they must take an Objective Structured Clinical Examination, and complete a residency program. Evaluation is conducted on continual performance until the period of supervised practice is complete. Successful IMGs are granted a license at this point, but may be required to participate in mentorship and multi-source feedback programs.

Achieving all of these milestones involves numerous professional and personal challenges. IMGs need to adapt to methods of learning, clinical procedures and knowledge, health care systems, patient expectations, collegial and supervisory relationships, team approaches, communication styles, legislative acts, legal issues, college requirements, and language improvement [8]. Additional difficulties include finding employment, and integrating into the professional role. They also bear the costs of becoming licensed, must access information on licensure procedures, and have the burden of studying for and passing exams [9,10].

The transition in medical practice from one country to another may be characterized as a “disorienting journey” [11]. Uncertainty about obtaining a license and restrictions to practice may be upsetting, but it may also promote one’s exploration of personal and professional identity. This may actually present an opportunity for IMGs to further their resolve to become recognized as doctors in a country other than where they were educated, or challenge their beliefs about their desire to do so. It can, moreover, be investigated empirically.

### Ego identity status

The process of developing a personal sense of identity is known as ego growth, or ego identity [12,13]. One’s selfhood or individuality emerges as a result of social interactions within the family and larger society based on norms of behaviour [14]. According to Damon [15], p.5, “The individual can only construct the self in the context of relations with others, but at the same time, the individual must step beyond the confines of those relations and forge a unique destiny.” Marcia’s [16,17] approach to ego identity was to examine how individuals make decisions about their religious and political beliefs, friendships, dating, recreational activities, and careers. Applied to career decision-making, he identified four pathways to forming a vocational identity (i.e., identity statuses), which are based on levels of exploration and commitment. There are two statuses of high commitment: Identity-achieved refers to people who have sought various career opportunities before making a commitment to pursue a specific career. Foreclosed refers to people who have also made a strong commitment to a career but have done so without having explored other careers. This commitment may occur in response to their perceptions of pressure by family members to choose a specific career. There are also two statuses of low commitment: Identity-moratorium is classified when people “try out” different careers without a commitment to any particular one. Finally, diffusion represents non-committal actions and little examination of career choices. High and low levels of commitment and exploration can be analyzed to determine how people make a decision about a career.

Despite decades of research on the theoretical importance and practical value of Marcia’s ego identity conceptualization [18], it has not been systematically applied to understanding how people choose a career in medicine. Two noteworthy studies have focused on ego identity development in medical students. In one study, Niemi [19] classified half of the participants into two of the four identity statuses to determine how pre-clinical students selected a medical specialization (the remaining students were not classified). Specifically, about a quarter had actively considered many specializations and made a firm decision on one (achieved). A similar number of students had neither considered nor selected a specialization (diffused). In another study conducted by Beran et al. [20], all sampled students in their clinical year of medical school were assessed for identity status. Almost half of the clinical students were achieved and half were foreclosed, with only 1% in the moratorium category, and none diffused.

The ego identity status of IMGs has not yet been examined. It is not known the extent to which they explore and commit to medicine as a career choice when facing immense personal and professional challenges. The psychological adaptation IMGs must make is critical to establishing personal and professional competence as a physician [21]. This process, however, has not been systematically studied. The purpose of the present study was to identify the ego identity status of IMGs, and whether it differs between CIMGs and non-CIMGs.

## Methods

### Participants

IMGs registered with the Alberta International Medical Graduate Program responded to posters and emails requesting volunteers for this study. Of the 50 physicians who responded to the request, 46 (92.00%) agreed to be interviewed. They were administered informed consent, notifying them of the purpose of the interviews. More than half (n = 26) of them were non-CIMGs (M age = 39.7, SD = 6.4 years) from 12 countries (17 women, 65% and 9 men, 35%; mean years post graduate training = 5.63). English was the language of medical training for 43.7% of these participants, several other languages account for the remainder with Spanish (13.6%), Russian (9.2%), Farsi (7.6%), Chinese (5%), Mandarin (3.4%), Romanian (3.4%) and other (14.1%). Almost all of the respondents (91.1%) perceived themselves as fluent English language users. The 20 CIMGs (M age = 32.5, SD = 3.8 years) had graduated from a non-Canadian medical school (14 women, 70% and 6 men, 30%; mean years post graduate training = 3.45). All had received their pre-medicine education from a Canadian university and were fluent English language users.

### Assessments

Five questions about career decision-making were developed through expert input and discussion and then employed in a semi-structured interview: “When did you first become interested in medicine?”, “When in university, either before or after entering the study of medicine, did you ever consider other areas besides medicine?”, “Growing up, did your parents have any plans for you for when you became an adult?”, “Have you ever pursued other types of work?”, and “How long are you prepared to work towards becoming fully licensed?”

### Procedures

We employed Silverman’s [22] procedures as valid and reliable methods for managing qualitative data. First, all interviews were audio recorded and transcribed verbatim. Second, InVivo was used to identify key words and phrases relevant to the codes established by Marcia et al. [23]. Two raters (TNB and CV) then classified each participant separately into one of the four identity statuses. Foreclosed was identified when participants decided on a career in medicine in their childhood, and did not explore any alternate careers before becoming a doctor. For example, one participant stated, “*I first became interested in medicine when in Grade 3 - I witnessed my grandfather’s death, he went into cardiac arrest and he was not able to receive medical care. I decided I would become a doctor and serve my family, community and nation.*” Achieved was classified for participants who decided to become a doctor when in adulthood, and who had pursued other career options outside of medicine. An exemplar response is, “*In high school I did not think I would like to work in a hospital. Late in my undergrad I thought about medical school and then in graduate school I started to think about it more seriously. My main interest was academia. I then worked for an engineering company doing product testing because of my biomechanics background. I thought about staying on with this. I also thought about going straight into epidemiology through graduate school rather than through medicine.*

When asked about how long they would pursue licensing, all participants indicated they would pursue this goal as long as it takes. This high level of commitment is confirmed by the participants’ initiation of the licensure process through registration and fee payment. They were, thus, all classified as showing a high degree of commitment to becoming licensed as a doctor in Canada. It is unlikely, therefore, that any of the IMGs in our sample are in the identity statuses of moratorium or diffusion. Two raters independently coded the identity status of participants as foreclosed or achieved with an initial result of 91.30% agreement and 100% agreement after discussion. Ethics approval was obtained from the University of Calgary Ethics Review Board before collecting data.

## Results

To determine whether ego identity status differs between CIMGs and non-CIMGs, event contingency analysis was conducted with responses from all five questions. There was a significant difference in the proportion of IMGs classified as achieved and foreclosed according to Fisher’s exact test (1) = 18.79, *p* < .0001. A total of 55% of CIMGs were classified as achieved, and 45% were classified as foreclosed. All non-CIMGs, in contrast, were classified as foreclosed (Table 1). There were no sex-differences in identity status for CIMGs or non-CIMGs.

**Table 1:** **Proportion of Canadian and non-Canadian IMGs classified by Ego Identity Status (*n*** **= 46)**

	**CIMG (*n* = 20)**	**Non-CIMGs (*n* = 26)**
Ego status		
Achieved	11 (55%)	None (0%)
High commitment/High exploration	*I first became interested in medicine as an adult - two years before applying to medicine. I did a business degree when I was in engineering.*	
Foreclosed	9 (45%)	26 (100%)
High commitment/Low exploration	*I was always committed to medicine. If I didn’t get into medicine I would have got further science degrees and then applied to medicine again. I never did any long term work.*	*When I was two or three years old my parents told me I would be a doctor, and I believed them. I am working to do what it takes to become a doctor so I am not spending time with a job or courses outside of medicine.*

Note: Fisher’s exact test (1) = 18.79, *p* < .0001.

## Discussion

Ego identity status of IMGs differs between CIMGs and non-CIMGs. That is, just over half of those IMGs who were Canadian citizens and obtained a medical degree in a foreign country were classified as achieved, and just under half were classified as foreclosed. Those IMGs who were citizens in a foreign country where they obtained their medical degree before moving to Canada, in contrast, were all classified as foreclosed. In addition, all IMGs demonstrated their firm commitment to enter practice in Canada.

Our study shows that Canadian and non-Canadian IMGs have undergone distinct career-decision making processes to become a doctor. While many IMGs in both groups foreclosed on their careers, other CIMGs had also considered a variety of careers before committing to medicine. These results are consistent with Beran et al. [20] who reported that half of their sample of Canadian students in clerkship identified a foreclosed status, and half an achieved status. Perhaps having spent more of their lives in Canada, compared to non-CIMGs, the emphasis on individualism in the Canadian culture to make choices apart from others’ suggestions influenced their independence in choosing their own career. IMGs from other countries with a collectivistic culture may have been strongly influenced by their parents who encouraged them to become doctors. Indeed, this was apparent in some of their statements about their parents strongly encouraging them to choose medicine. In addition, the education system of entering medical school after a non-medical undergraduate degree in Canada provides opportunities for taking courses in areas related to or distinct from medicine. These educational experiences may allow consideration of careers other than medicine. Conversely, most non-CIMGs entered medicine directly from high school and, therefore, had fewer opportunities to explore other career options. In addition, in some countries there may be restrictions such as age limits for the applicant (e.g., age 25 years), a maximum number of attempts at competitive entrance examinations, and the requirement of specific content expertise (e.g., zoology, botany, physics and chemistry).

IMGs face many obstacles to licensure: It is a complex and lengthy process with a low rate of residency matches (19.8%) for IMGs [24]. The prospect of the inability to obtain a license to practice medicine for those IMGs who have foreclosed on their career, with no prospects or even desire for another career, may explain why they are at risk of feeling alienated, isolated, and angry [9]. Nevertheless, if they develop the resiliency to overcome these challenges they may actually experience less fatigue, higher self-esteem, and greater personal growth than non-IMGs [25]. Support from colleagues who are now in practice in Canada, access to information about the licensure process in Canada before the decision is made to move to Canada, and workshops on managing the process of application will assist IMGs in overcoming the many obstacles they will face.

### Limitations

Several limitations need to be considered. First, only those IMGs who were applying for licensure were included in our study. Those who had not begun this process may be classified in other ego identity statuses according to differing levels of commitment. Second, retrospective reporting in this study relied on participants remembering career-related experiences as early as childhood, and these descriptions may be inaccurate. Also, their current career transition may have also influenced their descriptions of their earlier decision-making in becoming a doctor. These biases, however, are likely to have occurred in both groups, thereby not accounting for the difference in status between groups. Finally, this study is limited to a convenience sample of only those IMGs who volunteered to participate and who were seeking licensure in one province within Canada - results may not represent IMGs in other provinces or countries as licensing procedures may somewhat vary and differentially affect the professional identity of IMGs. In addition to replication, it is recommended that future research examine the socio-economic background of the participants to obtain a broad profile of the IMGs.

## Conclusions

The preponderance of foreclosure in choosing medicine as a career is unsurprising given that an extremely high level of commitment is necessary to become a doctor, thus allowing limited time to pursue and even consider other careers. The obstacles of licensing, such as difficulty passing exams, obtaining residency positions, or securing a clinical position, directly challenge the sense of identity as “doctor”, particularly for those who foreclosed on this career decision.

## Abbreviations

IMG, International Medical Graduates; CIMG, Canadian International Medical Graduates.

## Competing interests

The authors declare that they have no competing interests.

## Authors’ contributions

TNB and CV prepared the research assistants to conduct the interviews, analyzed the data, and wrote the findings. EV, SF DW and DL helped to develop the interview protocols, conducted the interviews, compiled the data and edited the manuscript. All authors read and approved the final manuscript.
